# The current status of 5-ALA fluorescence-guided resection of intracranial meningiomas—a critical review

**DOI:** 10.1007/s10143-015-0615-5

**Published:** 2015-03-05

**Authors:** Arash Motekallemi, Hanne-Rinck Jeltema, Jan D. M. Metzemaekers, Gooitzen M. van Dam, Lucy M. A. Crane, Rob J. M. Groen

**Affiliations:** 1 University of Groningen, Groningen, The Netherlands; 2 Department of Neurosurgery, University Medical Center Groningen, University of Groningen, PO Box 30.001, 9700 RB Groningen, The Netherlands; 3 Department of Surgery, University Medical Center Groningen, Groningen, The Netherlands; 4 University Medical Center Groningen, Groningen, The Netherlands

**Keywords:** 5-ALA, 5-aminolevulinic acid, Intracranial meningioma, Intraoperative fluorescence, Fluorescence-guided surgery, Fluorescence-guided resection

## Abstract

Meningiomas are the second most common primary tumors affecting the central nervous system. Surgical treatment can be curative in case of complete resection. 5-aminolevulinic acid (5-ALA) has been established as an intraoperative tool in malignant glioma surgery. A number of studies have tried to outline the merits of 5-ALA for the resection of intracranial meningiomas. In the present paper, we review the existing literature about the application of 5-ALA as an intraoperative tool for the resection of intracranial meningiomas. PubMed was used as the database for search tasks. We included articles published in English without limitations regarding publication date. Tumor fluorescence can occur in benign meningiomas (WHO grade I) as well as in WHO grade II and WHO grade III meningiomas. Most of the reviewed studies report fluorescence of the main tumor mass with high sensitivity and specificity. However, different parts of the same tumor can present with a different fluorescent pattern (heterogenic fluorescence). Quantitative probe fluorescence can be superior, especially in meningiomas with difficult anatomical accessibility. However, only one study was able to consistently correlate resected tissue with histopathological results and nonspecific fluorescence of healthy brain tissue remains a confounder. The use of 5-ALA as a tool to guide resection of intracranial meningiomas remains experimental, especially in cases with tumor recurrence. The principle of intraoperative fluorescence as a real-time method to achieve complete resection is appealing, but the usefulness of 5-ALA is questionable. 5-ALA in intracranial meningioma surgery should only be used in a protocolled prospective and long-term study.

## Introduction

Meningiomas are the second most common primary tumors affecting the central nervous system, accounting for more than 35 % of the primary brain tumors in adults [1]. The vast majority of meningiomas are slow-growing, benign (non-cancerous) tumors, although certain subtypes are more aggressive than others, and benign does not mean that they are without risk. Depending on its size and location, a benign meningioma can cause significant problems to the patient, become life-threatening, and be extremely difficult to treat. Originating from the dural covering of the brain, meningiomas are classified based on the site of origin, the involvement of adjacent tissues (venous sinuses, bone, brain, and nerves), and histological grade. The majority of meningiomas fall into histopathological classification WHO grade I, whereas atypical meningiomas (WHO grade II), and malignant or anaplastic meningiomas (WHO grade III) occur less frequently and constitute approximately 5–7 % of all meningiomas [2]. Being diagnosed with an intracranial meningioma means a reduction of life-expectancy to the patient, which is largely caused by tumor recurrence or outgrowth of tumor remnants, irrespective of the WHO grade. Even tumors that are (reported to be) resected completely may recur, which makes the operative treatment of every intracranial meningioma a challenge for the surgeon.

Incompletely resected tumors and high-grade lesions are frequently additionally treated with fractionated radiotherapy or stereotactic radiosurgery, and sometimes with hormonal- and immunotherapy [3–7]. Recurrences that occur after adjuvant/additional treatment often leave the surgeon with reoperation as the final and only option left for the patient. Computer-assisted operative systems (neuronavigation) have the potential to assist the surgeon in maximizing the extent of surgical resection, but there are limitations of this technique due to the phenomenon of “brain shift,” and the absence of real-time feedback [8–14]. To aid the intraoperative discerning of tumor from normal brain tissue, dural vessel involvement, bone invasion, and brain invasive growth, real-time fluorescence imaging was introduced, aiming to overcome the aforementioned problems. Although the first attempt of visualizing brain tumors using a fluorescent dye (fluorescein) was performed in 1948 by Moore and Peyton [15], it took almost 50 years until the principle was applied in a larger surgical setting [16]. Currently, 5-aminolevulinic acid (5-ALA) is used to aid intraoperative identification of malignant gliomas [17]. Since its introduction, 5-ALA fluorescence has been applied in a range of different types of CNS tumors, such as ependymomas, hemangioblastomas, metastatic brain tumors, and also in intracranial meningiomas. A number of authors have tried to outline the merits of 5-ALA in meningioma surgery, and have published their experience in this field [18–24], advocating its use in current neurosurgical practice. Nevertheless, the reliability of 5-ALA in meningioma surgery has not been established yet. Items such as specificity and sensitivity have not been solved, and the influences of histopathological grade, previous treatment like radiotherapy, radiosurgery, and medication (steroids, hormonal treatment, and chemotherapy) on the ability of meningioma cells to produce fluorescence remain unknown. In this review, we summarize the reports and clinical studies that have been published about the application of 5-ALA in intracranial meningioma surgery, and we report two illustrative cases of our institutional experience, in order to define the status quo of this technique in current intracranial meningioma surgery.

### 5-Aminolevulinic acid

5-Aminolevulinic acid is an indirect fluorophore and a natural biochemical progenitor of hemoglobin. Administered orally, 5-ALA is resorbed through the upper intestine into the blood, where it passes the blood–brain barrier [25].

It provokes the synthesis of protoporphyrin IX (PpIX) and is considered an endogenous photosensitizer. Excited with fluorescence excitation light (400–440 nm), PpIX emits red light energy of 635 nm, which is visible for the human eye. Although 5-ALA accumulates fairly selective in neoplasms, this process is not entirely tumor-specific as non-malignant tissue, i.e., brain parenchyma, the subependymal zone, and choroid plexus also show PpIX accumulation [26–28]. Vice versa, several studies and our institutional experiences show that not all tumors or tumor parts become fluorescent with 5-ALA [18, 20, 21, 23]. Moreover, the degree of heterogeneity in fluorescence for different tumor grades remains a major concern [29].

## Methods

### Search terms

PubMed was used for search tasks, using the key words “intracranial meningioma,” “5-aminolevulinic acid,” “5-ALA,” and “intraoperative fluorescence-guided resection.” There were no limitations with regard to publication date and language.

## Results

### Literature search

The literature search identified 11 publications, all in English, that reported of 5-ALA-assisted intracranial meningioma surgery. These papers are the subject of the present review.

### 5-ALA fluorescence-guided resection of intracranial meningiomas in literature

Several studies (*n* = 11) report of 5-ALA-assisted meningioma surgery. An overview is presented in Table 1.

**Table 1:** Data of the studies included in this review in chronological order

Study	Study description	Number of cases	Sensitivity (%)	Specificity (%)	WHO grade			RT
					I	II	III	
Kajimoto et al. [18]	24 patients. Four cases (3 grade I, and 1 grade II) did not show any fluorescence. No correlation between MIB-1 and fluorescence pattern was found.	24	83	100	18	4	2	Unknown
Morofuji et al. [19]	83-year-old female patient with a WHO grade II meningioma, which was clearly discernable with 5-ALA.	1	n. a.	100		1		No
Collucia et al. [20]	33 patients. Two cases (grade I) did not show any fluorescence and did not have a high MIB-1 or MI.	33	94	100	26	6	1	Unknown
Whitson et al. [21]	53-year-old female patient with a recurrent meningioma. Minimal background fluorescence of uninvolved dura was noticed. Part of the tumor signal did not rise above background fluorescence levels.	1	n.a.	100	1			Yes
Valdes et al. [22]	Six patients with WHO grade I and II meningiomas. Qualitative (visual) and quantitative (probe) fluorescence were compared revealing a higher accuracy and sensitivity of the quantitative fluorescence.	6	100 (probe) 80 (visual)	93	6 grade I and II			Unknown
Bekelis et al. [23]	52-year-old female patient with a grade I meningioma and intraorbital invasion. Qualitative (visual) and quantitative (probe) fluorescence were compared. Probe fluorescence yielded 100 % sensitivity.	1	100 (probe) 80 (visual)	Not provided	1			Unknown
Chae et al. [24]	69-year-old male patient with a meningioma in the Sylvian fissure with strong attachment to the Sylvian vein.	1	n.a.	100	1			Unknown
Cornelius et al. [30]	A 65-year-old female patient with an olfactory groove meningioma with infiltration of dura and bony scull base.	1	n.a.	Not provided	Not provided			Unknown
Cornelius et al. [31]	31 patients. Two cases (grade I) did not show any fluorescence, whereas 17 cases (14 grade I) showed “low” and twelve cases (three grade I) “high” fluorescence. Highly significant correlation between WHO grade and fluorescence intensity was found.	31	94	100	19	8	4	38 % of grade II 100 % of grade III
della Puppa et al. [32]	12 patients affected by bone-invading meningiomas (7 with skull base and 5 with convexity meningiomas). Positive and negative predictive values were 100 and 82.93 %, respectively. Their findings suggest that hyperostotic bone might influence the sensitivity of 5-ALA fluorescence.	12	100	89	10	2		Unknown
Valdes et al. [33]	15 patients. Two of the patients harbored recurrent meningiomas (grade II). Three cases (20 %) did not show any fluorescence. Quantitative fluorescence was measured in ten patients with grade I meningiomas. No statistical significance was found between WHO grade and fluorescence.	15	94 (probe) 80 (visual)	81	11	4		Unknown
Total	126							

*RT* previous radiotherapy treatment, *MIB-1* proliferation index, *MI* mitotic index, *n.a*. not applicable

Kajimoto et al. [18] published the first report about 5-ALA fluorescence-guided resection in meningiomas in 2007. Fluorescence-guided resection was performed in 24 non-consecutive patients revealing 18 benign, 4 atypical, and 2 anaplastic meningiomas. 5-ALA-induced fluorescence had a sensitivity of 83 % with a specificity of 100 %. Interestingly, 3 of 18 (16.7 %) benign meningiomas and 1 of 4 (25 %) atypical meningiomas did not show fluorescence at all [18]. Intraoperatively, six cases showed 5-ALA fluorescence of the dura. However, dura infiltration with tumor cells was confirmed in five of these cases. In all 24 cases, no strict correlation between cell proliferation and fluorescence pattern was found. Furthermore, grade of malignancy and fluorescence intensity were not related. In order to demonstrate the applicability of the 5-ALA technique, the authors illustratively discuss the case of a gross-total resection of a recurrent large atypical left convexity meningioma in a 65-year-old patient. Exploration for residual tumor was performed using 5-ALA assistance. In this case, 5-ALA selectively marked tumor tissue, enabling tumor resection with free resection margins and recurrence-free follow-up until 2.5 years after surgery [18].

Coluccia et al. [20] published the largest series thus far in 2010. They investigated the utility of 5-ALA for the resection of intracranial meningiomas in 33 consecutive patients. Histopathological grading revealed 26 benign (WHO grade I), 6 atypical (WHO grade II), and 1 anaplastic (WHO grade III) meningioma. The authors report 100 % sensitivity of 5-ALA fluorescence with a specificity of 94 %. Two cases did not show any fluorescence. Both were grade I meningiomas with low proliferation (MIB-1) and mitotic index (MI). In patients with positive fluorescence, no correlation of fluorescence and histopathological grade was found [20]. In 2011, Morofuji et al. [19] presented the case of an 83-year-old female patient. Previously obtained data using CT and MRI revealed a cranium invading, rapidly growing tumor, which proved to be an atypical meningioma (WHO II). During surgery, a highly fluorescent tumor was seen, whereas the surrounding dura mater appeared to be signal free. Tumor invasion into the diploë to the inner table at the stump of the upper orbital margin was not detectable with the standard surgical microscope, but clearly discernable with 5-ALA. The authors report 100 % correlation of histological results with intraoperative findings. They also found a higher accuracy using 5-ALA-induced fluorescence than contrast enhancement from previously obtained MRI scans. Consequently, the authors concluded that 5-ALA-assisted meningioma resection is not only superior to naked eye evaluation but also has the potential to accurately distinguish tumor-infiltrated bone and dura mater from healthy tissue [19].

Whitson et al. [21] report similar results in their case study of a 53-year-old female patient with a recurrent meningioma, albeit with slight differences. Initially, a WHO grade II meningioma was resected and the first recurrence was treated with radiotherapy. Routine follow-up MRI scans indicated recurrence of what appeared to be a more aggressive neoplasm along the falx, extending into both frontal lobes. During 5-ALA-assisted resection, two large nodules were found along the falx, which showed inhomogeneous fluorescence. Neither naked eye nor fluorescence evaluation showed involvement of surrounding brain parenchyma. However, minimal background fluorescence of uninvolved dura was noticed microscopically. Interestingly, different parts of the tumor signal did not rise above background fluorescence levels. Histopathological evaluation of the tumor discovered a WHO grade I meningioma with a very low proliferative index. Despite of this, the authors concluded that fluorescence yields a clear delineation of tumor tissue, although histological results did not correlate with their findings, thus ignoring the inhomogeneous or even absent fluorescence in certain parts of the tumor [21].

In 2012, Chae et al. [24] discussed the case of a 69-year-old male patient with a meningioma in the Sylvian fissure with strong attachment to the Sylvian vein. Due to the complicated location of the tumor and involved normal brain structures, 5-ALA-induced fluorescence was used to achieve resection of residual tumor tissue and prevent structural damage of the surrounding tissue. Pathological examination revealed a benign meningioma (WHO I) [24].

Cornelius et al. [30] evaluated 5-ALA and 18F-FET-PET as metabolic imaging tools for resection of a recurrent olfactory groove meningioma. Surgery was upon others handicapped by extensive growth of the tumor in surrounding tissue, delineation of pathological dura, infiltration of bony skull base, and scarring due to prior surgeries. In the reported case, 5-ALA fluorescence helped to dissect the adherent interface between tumor and brain, and to distinguish meningioma tissue from swollen mucosa and thus enabled straightforward dissection. However, for intraoperative assessment of bony and dural infiltration, FET-PET was used.

A larger study of Cornelius et al. [31] evaluated the impact of 5-ALA fluorescence-guided surgery on the extent of resection of meningiomas, with special regard to high-grade tumors. The authors investigated the potential of 5-ALA-guided surgery for discriminating different WHO grades intraoperatively, and analyzed whether fluorescence had an impact on the extent of resection and influenced surgical strategy. Thirty-one meningioma patients between 2008 and 2014 were operated using 5-ALA assistance. The investigated group consisted of 19 WHO grade I, 8 WHO grade II, and 4 WHO grade III tumors. Seventy-five percent of high-grade meningiomas (grades II and III) were recurrences at the time of surgery, of which 58 % had been previously treated with radiotherapy.

Two cases (grade I) did not show any fluorescence, whereas 17 cases (14 grade I) showed “low” and 12 cases (3 grade I) “high” fluorescence. Highly significant correlation between WHO grade and fluorescence intensity was found. 5-ALA improved the extent of resection in 19 % of the benign meningiomas and in 75 % of high-grade lesions compared to white light, especially in infiltrated brain, around brain vessels, and cranial nerves. However, 5-ALA-assisted surgery did not influence surgical strategy.

Recently, Della Puppa et al. [32] assessed the role of 5-ALA fluorescence in guiding the resection of bone-invading meningiomas in 12 patients (8 females and 4 males) delivering 98 bone samples. Meningioma tissue as well as bone invasion showed overall bright fluorescence. Bone invasion was confirmed histologically in 100 % of the fluorescent samples. However, their work showed that non-fluorescent bone can contain areas of meningioma invasion in approximately 13 % of the investigated cases. Their findings suggest that hyperostotic bone might influence the sensitivity of 5-ALA fluorescence. Postoperatively, two cases of non-fluorescent hyperostosis were reported, where the existence or absence of potential residual tumor tissue was indeterminable.

The previous reports [18–21, 24, 30–32] discussed the qualitative (visual) usefulness of 5-ALA as an intraoperative imaging tool for the resection of intracranial meningiomas. The following reports [22, 23, 33] also take the quantitative protoporphyrin IX concentrations into account.

Valdes et al. [22] compared the sensitivity and specificity of visual fluorescence of PpIX with its imperceptible quantitative concentrations in several brain tumors, using a specially designed probe for intraoperative fluorescence and white light reflectance measurement. Fourteen patients with low-grade glioma, high-grade glioma, meningioma, and metastases were included. Six of these patients harbored WHO grade I or II meningiomas. Quantitative probe fluorescence in meningiomas was associated with a higher sensitivity (99 %) than visual fluorescence (80 %), with an overall specificity of 93 % [22].

A more recent study of Valdes et al. [33] evaluated the use of qualitative and quantitative 5-ALA assistance in 15 meningioma patients. They investigated quantitative fluorescence levels in ten benign cases highlighting the importance of this approach in detecting otherwise non-visible but diagnostically relevant fluorescent levels of PpIX concentrations (0.010 mg/ml). They detected significant levels of PpIX in 69 % of histologically confirmed instances of tumor tissue with no visible fluorescence.

Bekelis et al. [23] report of a 52-year-old female patient with a WHO grade I meningioma with intraorbital invasion. This study also evaluated qualitative and quantitative fluorescence. Qualitative assessment of visible fluorescence was performed using naked eye evaluation. An intraoperative probe for in situ fluorescence detection was used for quantitative assessment of varying PpIX concentrations in tumor and healthy tissue. Intraoperative probe fluorescence yielded 100 % sensitivity and as such proved to be superior to visible fluorescence, which had a sensitivity of 80 % in detecting tumor tissue. Furthermore, histopathologically confirmed tumor tissue showed no visible levels of fluorescence but did accumulate significant PpIX concentrations (>0.10 mg/ml) [23]. The authors conclude that quantitative fluorescence using an intraoperative fluorescence probe may be used as an addition to standard surgical and fluorescence-guided resection, especially in meningiomas with difficult surgical accessibility or heterogeneous fluorescence pattern [22, 23, 33]. This approach could maximize the effect of 5-ALA fluorescence guidance while minimizing potential collateral damage.

### Illustrative cases from our own institute

**Fig. 1:**
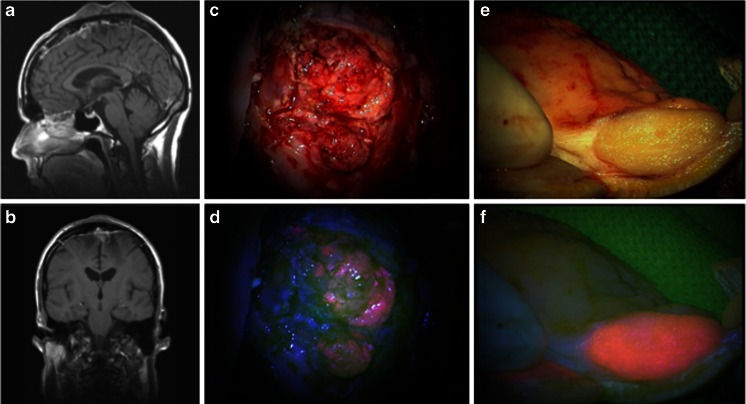
**a**, **b** MRI showing the third recurrence of a parasagittal meningioma with two prominent noduli involving the skin; **c**, **d** the intradural tumor part shows bright 5-ALA fluorescence; **e**, **f** the skin involving tumor noduli also show bright 5-ALA fluorescence 210 × 111 mm (72 × 72 DPI)

A 46-year-old male with a parietal meningioma (WHO grade I) underwent his first craniotomy in 2002. Due to a regrowth after 6 years (which at that time it appeared to be a WHO grade II meningioma), re-craniotomy and stereotactic radiosurgery (54 Gy) were performed. In the same year, routine follow-up showed regrowth with the now also involvement of the cranium. Histopathological analysis revealed an anaplastic meningioma (WHO grade III). In 2009, the tumor recurred, showing extracranial (cutaneous) and intracranial infiltration. Therefore, 5-ALA-assisted tumor resection was accomplished. Both intra- and extracranial tumor parts (both WHO grade III) showed bright fluorescence (see Fig. 1). The patient was additionally treated with stereotactic radiosurgery (20 Gy). In 2011 and 2012, two re-craniotomies were performed because of tumor growth, not far from the original operative area, both resulting into the resection of atypical meningiomas (WHO grade II).

**Fig. 2:**
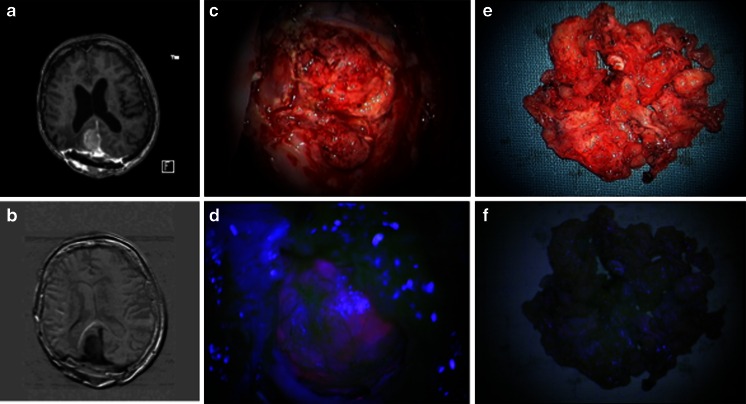
**a**, **b** MRI showing the fourth recurrence of a right sided occipital falx meningioma with tumor invasion of the skin; **c**, **d** intradural tumor part showing bright 5-ALA fluorescence; **e**, **f** subcutaneous tumor tissue does not show a fluorescent signal under violet-blue light 207 × 112 mm (72 × 72 DPI)

A 65-year-old female with a subtotal resection of an occipital falx meningioma (WHO grade I) in 1986 underwent re-craniotomy and radiotherapy treatment (60 Gy) in 2004 at our institution. Histopathological examination revealed an atypical meningioma (WHO grade II). In 2008, re-craniotomy was performed following tumor relapse (WHO grade II). Due to a next recurrence, 5-ALA-assisted surgery was performed in 2010, where the intracranial tumor was fluorescence-negative (meningioma WHO grade II). The next year, the tumor recurred, with an extension into the extracranial subcutaneous tissues. The intracranial part was compatible with a WHO grade II meningioma, while the extracranial tumor extension was consistent with a WHO grade III meningioma. 5-ALA-assisted resection showed no fluorescence of the extracranial tumor (WHO III), whereas the intracranial part (WHO II) showed bright fluorescence (see Fig. 2).

## Discussion

In this review, we summarized the application of 5-ALA-induced fluorescence as an intraoperative tool to maximize the resection of intracranial meningiomas. Furthermore, we discussed our institutional experiences with this technique presenting two illustrative cases.

On aggregate, a total number of 126 patients (not including our own two cases) with 5-ALA-assisted intracranial meningiomas resection are reported. All authors used a modified surgical microscope for fluorescence-guided visualization of the tumor and followed the protocol presented by Stummer et al. for the application of 5-ALA [17]. This protocol was designed for malignant glioma surgery and validation for meningioma surgery has not been done, as far as we know. We question the reliability of 5-ALA in intracranial meningioma surgery. As we showed, histologically identical meningiomas did not respond equally to 5-ALA administration. Furthermore, we observed a change in fluorescence response over time and a variation of fluorescence in different areas of the same tumor, which might have been caused by the influence of additional treatment (like radiotherapy or previous medical treatment) prior to reoperation of a recurrent meningioma.

Most studies [18–21, 24, 30–32] reported fluorescence of the main tumor mass with high sensitivity and specificity. In the majority of cases, fluorescence did lead to an extension of the resection and to the removal of additional tumor.

However, only Morofuji et al. [19] were able to consistently correlate resected tissue with histopathological findings. In three studies [22, 23, 33], state-of-the-art fluorescence imaging with 5-ALA was compared to quantitative probe fluorescence, resulting in a higher accuracy and sensitivity of the latter technique. However, the superiority of this approach above naked-eye evaluation of 5-ALA and its implementation in the operative routine need further investigation.

In meningioma surgery, there is no ultimate evidence regarding the appropriate resection margin to minimize recurrence [34–37]. Fluorescence imaging may help to more accurately determine whether excision margins are free of tumor cells. This requires validation of the exact detection threshold of the current intraoperative techniques. The findings in this review reveal that 5-ALA-induced fluorescence seems a promising approach for the detection of (remnant) meningioma cells. However, it lacks sufficient reliability regarding specificity [18, 21, 33] and histopathological accuracy to establish a consistent real-time assistance in meningioma surgery. In some cases, where fluorescence was reported, the difference between tumor and background fluorescence is hardly detectable with the naked eye [21, 24].

Our two illustrative cases revealed that tumor fluorescence under 5-ALA can be inconsistent and inhomogeneous, which limits the (presumed) benefits of fluorescence-guided surgery [18, 20, 21, 23, 30–33]. Pfisterer et al. [38] suggest that the genetic regional heterogeneity, that is often found in meningiomas, can be of high prognostic and diagnostic value. This could be an explanation for the inconsistency in fluorescence. Another explanation for the unspecific fluorescence pattern was provided by Masubuchi et al. [28], who demonstrated that PpIX is quickly excreted by meningioma cells into the extracellular space. Thus, depending on the threshold of the optical systems, non-malignant tissue might show false-negative fluorescence.

As stated previously, PpIX emits visible red light energy of 635 nm upon previous excitation. The eyes threshold for detection of this light depends on the retina, and its sensitivity/specificity for red light from, e.g., inborn retina diseases and age. These physiological limitations help to understand the different interpretation in visual fluorescence and emphasize the necessity for a more objective measurement of fluorescence (by quantitative biomarkers). However, even with the use of quantitative probe, the detection of tumor will depend on the concentration threshold for PpIX and on the experience of the surgeon.

In a recently published letter, Wilbers et al. [39] discuss their experience with an atypical meningioma. Using 5-ALA-induced fluorescence, the authors have been able to detect residual infiltration of the tumor in the dura and adjacent brain tissue. This can be essential regarding prognosis, regardless of the meningioma type. Moreover, the authors indicate the potential use of PpIX detection in adjuvant photodynamic therapy (PDT). This is worthy of further investigation as deep penetration of visible light (400–750 nm) is impeded, mainly due to autofluorescence by surrounding tissue and absorption by hemoglobin.

It is widely known that radiation therapy (RT) of the upper body, either as a direct effect of treatment or by incidental exposure, has an etiological effect on the development of brain tumors. Moreover, it is found that radiation-induced meningiomas express more atypical features and multiplicity, compared to non-radiation-induced meningiomas [40–43]. Our own experience is that recurrent meningiomas that have been treated with radiotherapy can show alteration in fluorescence. Most of the studies reviewed do not mention previous radiotherapy of their patients (see Table 1). However, such information is essential for the correct interpretation of 5-ALA-induced fluorescence. Notably, it is especially this category of patients (multiple craniotomies and radiotherapy for recurrent meningioma) that urges for the development of a tumor-specific tool to improve intraoperative tumor detection and a radical resection.

Based upon the above dilemmas, the question is how to proceed.

It is evident that there is no(t yet a) solid basis for the reliable application of 5-ALA in meningioma surgery. In our opinion, 5-ALA-assisted tumor resection should be protocolled and considered for all patients with primary and recurrent meningioma. Close and detailed follow-up is needed, also taking into account the complete patient history and previous medical treatment including radiation therapy and/or stereotactic surgery. Tumor specimen should be incubated and cultured, to allow for future tumor cell research and for the development of a tumor-specific intraoperative fluorescence techniques, as recently also has been suggested by Behbahaninia et al. [44].

Targeting of a tumor-specific biomarker with a fluorescent probe yields a high discrimination ratio between tumor and healthy tissue and as such, this technique has the potential to accurately identify tumor deposits and may be useful in the evaluation of tumor margins. 5-ALA (400–440 nm) operates in the visible light spectrum (400–750 nm); wavelengths in which the signal is partly limited by autofluorescence of background tissue and by absorption by hemoglobin. In contrary, tumor-specific imaging using fluorescent dyes in the near-infrared (NIR) range (750–1000 nm) yield better signal-to-background ratios [45]. Van Dam et al. [46] have successfully reported tumor-targeted fluorescence imaging in patients with ovarian cancer to detect metastatic tumor tissue. In this concept, it is essential to determine which biomarkers are upregulated most in the tumor cells. Those markers can serve as a target for a fluorescent imaging agent. Upon excitation by a laser, the reflected signal by the fluorescent agent can be detected by a sensitive camera system. Thus, identifying meningioma-specific biomarkers may be a challenging focus for future investigations. Image-guided resection of meningiomas could benefit from this concept. Fluorescent dyes with a NIR emission wavelength are likely to be more suitable for intraoperative imaging purposes due to their tumor-specificity. Thus, a tumor-specific approach would allow a more sensitive tumor delineation and assessment of tumor margins.

## Conclusion

It is challenging to draw definitive conclusions regarding the role of 5-ALA fluorescence-guided meningioma surgery from the present review. 5-ALA-assisted resection does not replace the resection of tumor under white light. Moreover, this new technique serves as additional information upon the surgeon’s experience, and his/her tactile and visual perception.

The following is a list of findings, based on our institutional experiences, and the status quo of this technique in the literature.

- Tumor cell fluorescence can appear in benign meningiomas (WHO grade I) as well as in high-grade lesions (WHO grade II and III) meningiomas.
- There seems to be no correlation between fluorescence intensity and proliferation rate (MIB-1 Labeling Index) or mitotic index (MI).
- Sensitivity and specificity rates vary between studies, but are generally high.
- Within the same tumor, fluorescence can be very different (heterogenic fluorescence), showing highly fluorescent parts, and parts that do not respond at all.
- The fluorescence response to 5-ALA administration in a recurrent meningioma of the same histological grade can differ from the response observed during the former resection, changing from “5-ALA positive” to “5-ALA negative,” or vice versa.

This review shows that 5-ALA, as a tool to guide resection of intracranial meningiomas, is still very experimental and should only be used in protocolled prospective studies. The superiority of 5-ALA-assisted resection of intracranial meningiomas regarding progression-free survival needs to be investigated in prospective cohort studies. However, the principle of fluorescence as a real-time method to assist the surgeon in achieving complete resection (and cure!) of benign intracranial tumors is very appealing. Besides, from the application of 5-ALA, the use of other real-time modalities, especially tumor-specific intraoperative fluorophores, are very worthy to be investigated.

## References

[1] Dolecek TA, Propp JM, Stroup NE, Kruchko C (2012). CBTRUS statistical report: primary brain and central nervous system tumors diagnosed in the United States in 2005–2009. Neuro–Oncol.

[2] Louis DN, Ohgaki H, Wiestler OD (2007). The 2007 WHO classification of tumours of the central nervous system. Acta Neuropathol.

[3] Whittle IR, Smith C, Navoo P, Collie D (2004). Meningiomas. Lancet.

[4] Chamberlain MC (2004). Intracerebral meningiomas. Curr Treat Options Neurol.

[5] McMullen KP, Stieber VW (2004). Meningioma: current treatment options and future directions. Curr Treat Options in Oncol.

[6] Norden AD, Drappatz J, Wen PY (2009). Advances in meningioma therapy. Curr Neurol Neurosci Rep.

[7] Sioka C, Kyritsis AP (2009). Chemotherapy, hormonal therapy, and immunotherapy for recurrent meningiomas. J Neuro-Oncol.

[8] Omay SB, Barnett GH (2010). Surgical navigation for meningioma surgery. J Neuro-Oncol.

[9] Willems PWA, van der Sprenkel JWB, Tulleken CAF (2006). Neuronavigation and surgery of intracerebral tumours. J Neurol.

[10] Ganslandt O, Behari S, Gralla J (2002). Neuronavigation : concept, techniques and applications. Neurol India.

[11] Paleologos TS, Wadley JP, Kitchen ND (2000). Clinical utility and cost-effectiveness of interactive image-guided craniotomy: clinical comparison between conventional and image-guided meningioma surgery. Neurosurgery.

[12] Barnett GH, Steiner CP, Weisenberger J (1995). Intracranial meningioma resection using frameless stereotaxy. J Image Guided Surg.

[13] Spetzger U, Laborde G, Gilsbach JM (1995). Frameless neuronavigation in modern neurosurgery. Minim Invasive Neurosurg.

[14] Albayrak B, Samdani AF, Black PM (2004). Intra-operative magnetic resonance imaging in neurosurgery. Acta Neurochir (Wien).

[15] Moore GE, Peyton WT (1948). The clinical use of fluorescein in neurosurgery; the localization of brain tumors. J Neurosurg.

[16] Stummer W, Novotny A, Stepp H (2000). Fluorescence-guided resection of glioblastoma multiforme by using 5-aminolevulinic acid-induced porphyrins: a prospective study in 52 consecutive patients. J Neurosurg.

[17] Stummer W, Pichlmeier U, Meinel T (2006). Fluorescence-guided surgery with 5-aminolevulinic acid for resection of malignant glioma: a randomised controlled multicentre phase III trial. Lancet Oncol.

[18] Kajimoto Y, Kuroiwa T, Miyatake S-I (2007). Use of 5-aminolevulinic acid in fluorescence-guided resection of meningioma with high risk of recurrence. Case Reprt. J Neurosurg.

[19] Morofuji Y, Matsuo T, Hayashi Y (2008). Usefulness of intraoperative photodynamic diagnosis using 5-aminolevulinic acid for meningiomas with cranial invasion: technical case report. Neurosurgery.

[20] Coluccia D, Fandino J, Fujioka M (2010). Intraoperative 5-aminolevulinic-acid-induced fluorescence in meningiomas. Acta Neurochir (Wien).

[21] Whitson WJ, Valdes PA, Harris BT, Paulsen KD (2011). Confocal microscopy for the histological fluorescence pattern of a recurrent atypical meningioma: case report. Neurosurgery.

[22] Valdés PA, Leblond F, Kim A (2011). Quantitative fluorescence in intracranial tumor: implications for ALA-induced PpIX as an intraoperative biomarker. J Neurosurg.

[23] Bekelis K, Valdés PA, Erkmen K (2011). Quantitative and qualitative 5-aminolevulinic acid-induced protoporphyrin IX fluorescence in skull base meningiomas. Neurosurg Focus.

[24] Chae MP, Song SW, Park S-H (2012). Experience with 5-aminolevulinic Acid in fluorescence-guided resection of a deep sylvian meningioma. J Korean Neurosurg Soc.

[25] Dalton JT, Yates CR, Yin D (2002). Clinical pharmacokinetics of 5-aminolevulinic acid in healthy volunteers and patients at high risk for recurrent bladder cancer. J Pharmacol Exp Ther.

[26] Olivo M, Wilson BC (2004). Mapping ALA-induced PPIX fluorescence in normal brain and brain tumour using confocal fluorescence microscopy. Int J Oncol.

[27] Tonn J-C, Stummer W (2008). Fluorescence-guided resection of malignant gliomas using 5-aminolevulinic acid: practical use, risks, and pitfalls. Clin Neurosurg.

[28] Masubuchi T, Kajimoto Y, Kawabata S (2013). Experimental study to understand nonspecific protoporphyrin IX fluorescence in brain tissues near tumors after 5-aminolevulinic acid administration. Photomed Laser Surg.

[29] Floeth FW, Sabel M, Ewelt C (2011). Comparison of (18)F-FET PET and 5-ALA fluorescence in cerebral gliomas. Eur J Nucl Med Mol Imaging.

[30] Cornelius JF, Slotty PJ, Stoffels G (2013). 5-Aminolevulinic acid and (18)F-FET-PET as metabolic imaging tools for surgery of a recurrent skull base meningioma. J Neurol Surg B Skull Base.

[31] Cornelius JF, Slotty PJ, Kamp MA (2014). Impact of 5-aminolevulinic acid fluorescence-guided surgery on the extent of resection of meningiomas—with special regard to high-grade tumors. Photodiagnosis Photodynamic Ther.

[32] Della Puppa A, Rustemi O, Gioffrè G (2014). Predictive value of intraoperative 5-aminolevulinic acid-induced fluorescence for detecting bone invasion in meningioma surgery. J Neurosurg.

[33] Valdes PA, Bekelis K, Harris BT (2014). 5-Aminolevulinic acid-induced protoporphyrin IX fluorescence in meningioma: qualitative and quantitative measurements in vivo. Neurosurgery.

[34] Borovich B, Doron Y (1986). Recurrence of intracranial meningiomas: the role played by regional multicentricity. J Neurosurg.

[35] Nakasu S, Nakasu Y, Nakajima M (1999). Preoperative identification of meningiomas that are highly likely to recur. J Neurosurg.

[36] Kinjo T, Al-Mefty O, Kanaan I (1993). Grade zero removal of supratentorial convexity meningiomas. Neurosurgery.

[37] Peyre M, Clermont-Taranchon E, Stemmer-Rachamimov A (2013). Miniaturized handheld confocal microscopy identifies focal brain invasion in a mouse model of aggressive meningioma. Brain Pathol.

[38] Pfisterer WK, Hank NC, Preul MC (2004). Diagnostic and prognostic significance of genetic regional heterogeneity in meningiomas. Neuro Oncol.

[39] Wilbers E, Hargus G, Wölfer J (2014). Usefulness of 5-ALA (Gliolan®)-derived PPX fluorescence for demonstrating the extent of infiltration in atypical meningiomas. Acta Neurochir.

[40] Preston-Martin S, Mack W, Henderson BE (1989). Risk factors for gliomas and meningiomas in males in Los Angeles county. Cancer Res.

[41] Taylor AJ, Little MP, Winter DL (2010). Population-based risks of CNS tumors in survivors of childhood cancer: the British childhood cancer survivor study. J Clin Oncol.

[42] Banerjee J, Pääkkö E, Harila M (2009). Radiation-induced meningiomas: a shadow in the success story of childhood leukemia. Neuro Oncol.

[43] Longstreth WT, Dennis LK, McGuire VM (1993). Epidemiology of intracranial meningioma. Cancer.

[44] Behbahaninia M, Martirosyan NL, Georges J (2013). Intraoperative fluorescent imaging of intracranial tumors: a review. Clin Neurol Neurosurg.

[45] Crane LMA, Themelis G, Pleijhuis RG (2011). Intraoperative multispectral fluorescence imaging for the detection of the sentinel lymph node in cervical cancer: a novel concept. Mol Imaging Biol.

[46] Van Dam GM, Themelis G, Crane LMA (2011). Intraoperative tumor-specific fluorescence imaging in ovarian cancer by folate receptor-α targeting: first in-human results. Nat Med.

